# A Comparison of the Anorectic Effect and Safety of the Alpha_2_-Adrenoceptor Ligands Guanfacine and Yohimbine in Rats with Diet-Induced Obesity

**DOI:** 10.1371/journal.pone.0141327

**Published:** 2015-10-27

**Authors:** Magdalena Dudek, Joanna Knutelska, Marek Bednarski, Leszek Nowiński, Małgorzata Zygmunt, Barbara Mordyl, Monika Głuch-Lutwin, Grzegorz Kazek, Jacek Sapa, Karolina Pytka

**Affiliations:** 1 Department of Pharmacodynamics, Jagiellonian University, Collegium Medicum, 9 Medyczna Street, PL 30–688 Kraków, Poland; 2 Department of Pharmacological Screening, Jagiellonian University, Collegium Medicum, 9 Medyczna Street, PL 30–688 Kraków, Poland; 3 Department of Pharmacobiology, Jagiellonian University, Collegium Medicum, 9 Medyczna Street, PL 30–688 Kraków, Poland; State University of Rio de Janeiro, Biomedical Center, Institute of Biology, BRAZIL

## Abstract

The search for drugs with anorectic activity, acting within the adrenergic system has attracted the interest of researchers. Partial α_2_-adrenoceptor agonists might offer the potential for effective and safe treatment of obesity. We compared the effectiveness and safety of α_2_-adrenoceptor ligands in reducing body mass. We also analyzed if antagonist and partial agonists of α_2_-adrenoceptor––yohimbine and guanfacine––act similarly, and determined which course of action is connected with anorectic activity. We tested intrinsic activity and effect on the lipolysis of these compounds in cell cultures, evaluated their effect on meal size, body weight in Wistar rats with high-fat diet-induced obesity, and determined their effect on blood pressure, heart rate, lipid profile, spontaneous locomotor activity, core temperature and glucose, as well as glycerol and cortisol levels. Both guanfacine and yohimbine showed anorectic activity. Guanfacine was much more effective than yohimbine. Both significantly reduced the amount of intraperitoneal adipose tissue and had a beneficial effect on lipid profiles. Decreased response of α_2A_-adrenoceptors and partial stimulation of α_2B_-receptors seem to be responsible for the anorectic action of guanfacine. The stimulation of α_1_-adrenoceptors by guanfacine is responsible for cardiovascular side effects but may also be linked with improved anorexic effect. α_1_-adrenoceptor blockade is connected with the side effects of yohimbine, but it is also associated with the improvement of lipid profiles. Guanfacine has been approved by the Food and Drug Administration (FDA) to treat hypertension and conduct disorder, but as it reduces body weight, it is worth examining its effectiveness and safety in models of obesity.

## Introduction

Obesity is affecting an increasing number of individuals in the human population [1]. It is estimated that over one billion people in the world of adults are overweight, and that obesity affects about 312 million of these people [2]. The World Health Organization has recognized obesity as an epidemic of the 21st century. Obese people more frequently suffer from cardiovascular diseases, diabetes, and diseases of the digestive system, and are at higher risk of developing cancer [3]. This shows the importance of conducting research leading to the discovery of a drug that will effectively reduce weight.

The idea to use α_2_-adrenoceptor antagonists, such as yohimbine, in the treatment of obesity has been considered by many researchers [4, 5]. However, their side effects were unacceptable and their effectiveness was insufficient. Yohimbine, a well-known α_2_-adrenoceptor antagonist, has an undesirable effect on blood pressure and heart rate [4, 6, 7], due to its effect on both central and peripheral adrenoceptors. Moreover, the higher doses of this drug, which were used for weight reduction, affected α_1_-adrenoreceptors as well. Since yohimbine at such doses can block both postsynaptic α_1_- and α_2_-adrenoceptors present in arterial vessels, there is a risk of a very significant drop in blood pressure.

In recent years, an increasing number of reports in the literature have suggested that the effectiveness of drugs such as sibutramine or bupropion in reducing weight is associated with the effect on α_2_-adrenoceptors [8, 9]. Therefore, inverse α_2_-adrenoceptor agonists may be useful in the treatment of obesity [9]. It is not surprising though that compounds possessing anorectic activity and acting within the adrenergic system have attracted the interest of researchers.

Guanfacine is a selective agonist of α_2_-adrenoceptors [10, 11], which in many experimental models acts differently than other agonists, e.g. clonidine [12, 13]. To the best of our knowledge, there are no studies in the literature concerning the anorectic effect of guanfacine in obesity models.

Guanfacine has been approved by Food and Drug Administration (FDA) to treat hypertension, and lately to treat conduct disorder in children and adolescents. As it shows weight reducing properties, we decided to examine its efficacy and safety in models of obesity and determine if it could be safely used in obese patients without clear cardiovascular problems.

Consequently, we have undertaken studies to determine the activity and safety of guanfacine in an experimental model of obesity in rats, analyzed its effect on α_2_-adrenoceptors and investigated which subtype of these receptors is most involved in reducing weight after chronic treatment.

In this paper, we compare the effectiveness of the anorexic action and safety of yohimbine––an α_2_-adrenoceptor antagonist––and guanfacine––a partial agonist of α_2_-adrenoceptor––in rats with diet-induced obesity and determine their effect on core temperature, blood pressure, heart rate and lipid and carbohydrate profiles, as well as glycerol and cortisol levels. We also compare their effect on the adrenoceptors, including α_2_-adrenoceptors subtypes. Moreover, we determine the effect of guanfacine on spontaneous activity and its lipolytic effect.

## Materials and Methods

### Drugs

Guanfacine, oxymetazoline, brimonidine, clonidine (Sigma-Aldrich, Germany), yohimbine (Tocris, Bristol, UK), heparin (Polfa Warszawa S.A., Warsaw, Poland), and thiopental (Sandoz International, France).

### Animals

The experiments were carried out on male Wistar rats (initial body weight 140–160 g, eight weeks of age, in the growth phase). The animals were housed in pairs in plastic cages in constant temperature facilities exposed to 12–12 light-dark cycle, water and food were available *ad libitum*. Control and experimental groups consisted of six to eight animals each. All experiments were conducted according to the guidelines of the Animal Use and Care Committee of the Jagiellonian University and were approved for realization (2012 and 2015; Poland; Permission No 54/2012 and 17/2015). All surgery was performed under thiopental anesthesia, and every effort was made to minimize suffering.

### Determination of the intrinsic activity of the α_2A_-adrenoceptor and α_2B_-adrenoceptor

Intrinsic assays of cells with stable expression of the α_2A_-adrenoceptor or α_2B_-adrenoceptor were performed using ready-to-use assay kits according the manufacturers' guidelines (Invitrogen Life Technologies and Perkin Elmer, respectively). Reference compounds were: brimonidine, oxymethazoline and clonidine.

### Obesity induced with a fatty diet and its effect on body weight, food and water intakes

Male Wistar rats (140–160 g) were pair-housed and fed a fatty diet consisting of 40% fat blend (Labofeed B with 40% lard, Morawski, Manufacturer Feed, Poland), with water available *ad libitum* for 10 weeks. Control rats were fed a standard diet (Labofeed B) for 10 weeks. Diet-induced obese rats were randomly divided into five equal groups ((the same mean body weight (b.w.) in each group)), and treated intraperitoneally (i.p.): 2 or 5 mg/kg/day yohimbine or 0.5 or 2 mg/kg/day guanfacine or vehicle (water– 0.3 ml), once daily, between 9:00 AM and 10:00 AM for 30 days. Intakes of food and water, and body weight were measured daily immediately prior to administration of drugs. On the 31st day, 20 minutes after intraperitoneal administration of heparin 1000 j/rat and thiopental (70 mg/kg b.w., i.p.), blood was collected from animals and was centrifuged for 10 minutes at 1500 rpm to separate plasma. Also, intraperitoneal fat was collected and weighed.

### The effect of guanfacine on locomotor activity during chronic treatment in diet-induced obese rats housed in pairs in home cages

The locomotor activity of rats treated with guanfacine was measured with a special RFID-system—TraffiCage (TES-Systems, Germany). The rats were housed in pairs in home cages with food and water available *ad libitum*. The animals were subcutaneously implanted with transmitter identification (RFID), which recorded the presence and time spent in different areas of the cage, and then grouped the data in a special computer program. The rats were treated intraperitoneally with guanfacine 2 mg/kg b.w./day or a vehicle—water 0.3 ml/kg (diet-induced obese-control group), once per day in the morning for 30 days. Locomotor activity was recorded for 24 hours after administration of the compound.

### Determination of the effect on blood pressure in rats after a single administration

Next, normotensive rats were anesthetized with thiopental (70 mg/kg) by i.p. injection. The left carotid artery was cannulated with tubing filled with heparin solution in saline to facilitate pressure measurements using Apparatus PowerLab 4/35 (ADInstruments, Australia). Blood pressure was measured: before intraperitoneal administration of guanfacine and yohimbine—time 0 min (control pressure) and during the next 90 min.

### The effect of guanfacine on lipid and carbohydrate profiles and glycerol levels in diet-induced obese rats

To determine the lipid profile and glucose level in the plasma, standard tests from Biomaxima S.A. (Poland) were used. To determine the glycerol level in the plasma, a Glycerol Colorimetric Assay Kit (Cayman, USA) was used.

### Effect of guanfacine on cortisol levels in diet-induced obese rats

To determine the cortisol level in the plasma, Cortisol Express EIA Kit (Cayman, USA) was used.

### Determination of lipolysis *in vitro*

#### Cell culture and differentiation

The 3T3-L1 cells were obtained from the ATCC (CL-173). The cells were thawed according to the manufacturer's protocol. 3T3-L1 cells were cultivated in Dulbecco's Modified Eagle's Medium (DMEM, ATCC) supplemented with 10% calf bovine serum (ATCC), 100 IU/ml penicillin (ATCC) and 100 μg/ml streptomycin (ATCC). The cells were seeded on 48-well culture plates at a density of 50 000 cells per well. To induce differentiation, 3T3-L1 were cultured for 2 days until a confluence was reached and then the culture medium was replaced with a fresh induction medium containing 10 μg/ml human recombinant insulin, 0.5 mM 3-isobutyl-1-methylxanthine and 1 μM dexamethasone. After 2 days, the medium was replaced with a differentiation medium containing 10 μg/ml human recombinant insulin only. Then, differentiation medium was replaced with a culture medium without differentiating factors. The culture medium was changed every 2 days. Adipocytes for experiments were used 18 days after the initiation of differentiation.

#### Compounds

All compounds were dissolved in dimethyl sulfoxide (DMSO) to obtain stock concentrations. From the stock solution, dilutions were prepared in phosphate buffered saline (PBS). The final concentrations of compounds were 50 μM, 10 μM and 1 μM. All experiments were performed in triplicates. The cells were incubated with the tested compounds for 24 hours.

#### Cell membrane damage

The bioluminescent ToxiLight bioassay (Lonza) is a highly sensitive cytotoxicity assay designed to measure cell membrane damage. After incubation of cells with the tested compounds, 10 μl of cell supernatant was deposited in a new 96-well plate. Then, 40 μl of the Adenylate Kinase Detection Reagent (AKDR) was added per well. After 5 minutes of incubation at 22°C, the luminescence intensity was measured in a multifunction plate reader (POLARstar Omega, BMG Labtech).

#### Cell viability

Cell viability was measured using PrestoBlue reagent (Life Technologies). After incubation of cells with the tested compounds, PrestoBlue reagent was added to each well of a microplate in an amount equal to one tenth of the volume of the remaining medium. After 20 minutes of incubation at 37°C, the fluorescence intensity (EX 530 EM 580 nm) was measured in the multifunction plate reader (POLARstar Omega, BMG Labtech).

#### Glucose utilization

An Amplex Red Glucose Kit (Life Technologies) was used to measure glucose utilization by 3T3-L1 after incubation with the tested compounds. To 10 μl of the tested sample 10 μl of 200 μM AmplexRed reagent (10-acetyl-3,7-dihydroxyphenoxazine) was added with 0.4 U/ml peroxidase (HRP), and 4 U/ml of glucose oxidase in 50 mM potassium phosphate buffer pH 7.4. After 30 minutes of incubation at 37°C, the fluorescence intensity (EX 530 EM 580 nm) was measured in the multifunction plate reader (POLARstar Omega, BMG Labtech).

#### Lipolysis

Lipolysis was evaluated by measuring the amount of glycerol released into the medium. The supernatants were collected, and the amount of glycerol was determined using the AdipoLyze Lipolysis Detection Kit (Lonza) according to the manufacturer’s protocol. After 2 hours of reagent incubation with the tested sample at 22°C, the fluorescence intensity (EX 520 EM 590 nm) was measured in the multifunction plate reader (POLARstar Omega, BMG Labtech).

### Effect of guanfacine and yohimbine on core temperature

Those animals with induced obesity were subcutaneously implanted with a DST micro-HRT heart rate logger, which simultaneously measured long-term heart rate and core temperature in the animal (Star Oddi, Island). The temperature was recorded both before and 24 hours after the intraperitoneal administration of guanfacine at the dose of 2 mg/kg b.w. Mercury software was used to collect and analyze the data. On the basis of this technology, measurements are made without the direct intervention of the researcher, so they do not cause disturbances evoked by stress in animals.

### Statistical analysis

The statistical calculations were carried out with the GraphPad Prism 6 program. Results are given as arithmetic means with SEM. The statistical significance was calculated using a one-way ANOVA with Dunnett's Multiple Comparison Test post-hoc or two-way ANOVA (Bonferroni test post-hoc) or Student’s t-test (when two groups were compared). Differences were considered statistically significant at: *P≤ 0.05, **P≤ 0.01, and ***P≤ 0.001.

## Results

### Intrinsic activity

The potency (pEC50) and intrinsic activity of oxymetazoline, brimonidine, guanfacine, clonidine and yohimbine at human cloned α_2_-adrenoceptors are shown in Table 1. Guanfacine and clonidine showed the properties of partial agonists of both α_2_-adrenoceptors subtypes (A and B). Guanfacine and clonidine activated subtype A of this receptor in concentrations approximately 50-fold lower than subtype B. Yohimbine showed antagonistic properties for both subtypes of α-adrenoceptors.

**Table 1:** Potency (pEC50) and intrinsic activity (relative to full agonist) of tested compounds at human cloned α_2_-adrenoreceptors.

Compound	α_2A_-adrenoceptor		α_2B_-adrenoceptor	
	pEC_50_	I.A.	pEC_50_	I.A.
**Brimonidine**	8.1 ± 0.05	1	NT	NT
**Oxymetazoline**	7.5 ± 0.02	0.6 ± 0.04	5.5 ± 0.02	1
**Guanfacine**	7.6 ± 0.03	0.8 ± 0.03	5.6 ± 0.04	0.5 ± 0.04
**Clonidine**	5.9 ± 0.03	0.7 ± 0.03	4.3 ± 0.21	0.3 ± 0.05
**Yohimbine**	8.3 ± 0.06	0—antagonist	7.8 ± 0.06	0—antagonist

I.A.–intrinsic activity; NT—not tested. pEC_50_ and I.A. were determined in functional assays (Tango, AequoScreen). Shown is the mean ± SEM (n = 3).

### Obesity induced with fatty diet

**Fig 1:**
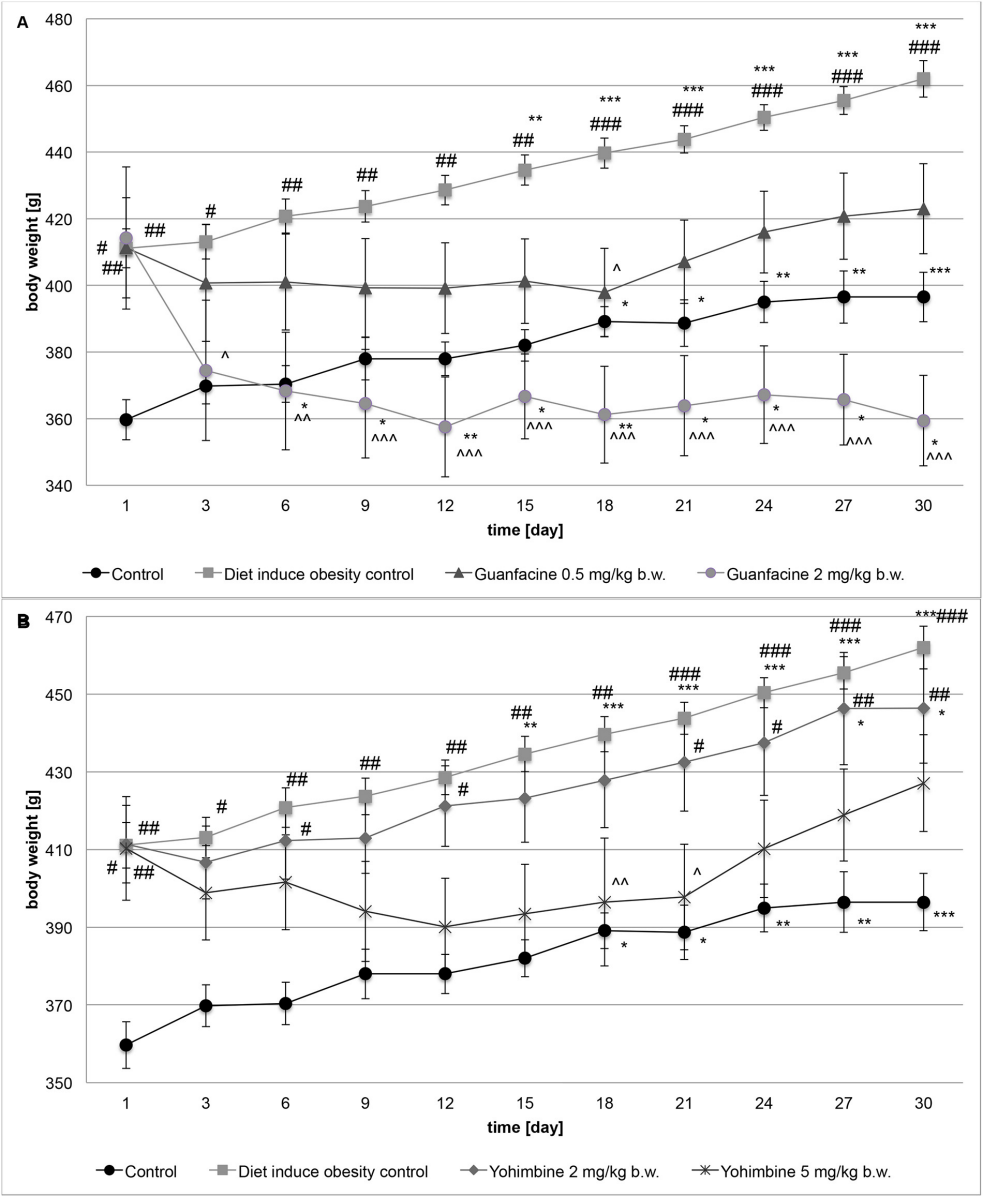
The effect of guanfacine (A) and yohimbine (B) on body weight. The change in body weight in control (standard diet) and diet-induced obese Wistar rats, treated for 30 days with the tested compounds; Control group—water; Diet-induced obesity control group—water; Yohimbine at 2 mg/kg b.w., i.p.; Yohimbine at 5 mg/kg b.w., i.p.; Guanfacine at 0.5 mg/kg b.w., i.p.; Guanfacine at 2 mg/kg b.w., i.p.; Significant to 1^st^ day: *P<0.05, **P<0.01, ***P<0.001 (ONE-WAY ANOVA) or significant to control group (water): **#**P<0.05, **##**P<0.01, **###**P<0.001 (TWO-WAY ANOVA) or significant to diet-induced obesity control group (water): **^**P<0.05, **^^**P<0.01, **^^^**P<0.001 (TWO-WAY ANOVA).

After 10 weeks of inducing obesity, the percentage of body weight difference between rats fed with standard food (control group) and rats fed a fatty diet (diet-induced obesity control group) was 14.29% (P<0.001). Table 2 shows the baseline body weight and body weight after 10 weeks of food consumption in these groups. Clearly, it can be concluded that obesity in this model was induced. For the next 30 days, the difference in body weight changes between groups remained practically unchanged, i.e. 15.82% (P<0.001). Results are shown in Fig 1.

**Table 2:** The effect of studied compounds on body weight, food and water intake.

	Control	Diet-induced obesity control
**Initial body weight, g**	151.7 ± 5.56	155.6 ± 2.06
**Final body weight, g**	359.7 ± 6.03	411.1 ± 5.85***
**Total food intake, kcal;**	4502.4 ± 364.40	7339.4 ± 898.70*
**Total food intake, g of food**	1608 ± 130.10	1334.4 ± 163.4
**Total water intake, ml**	2312.5	2013.9

Body weight, food intake, water intake during 10 weeks inducing obesity in Wistar rats on control food or diet-induced obesity, mean on one individual ± SEM. Treatment: Control group—water, Diet-induced obesity control group—water;

*Significant vs. control group, P<0.05;

***Significant vs. control group, P<0.001.

During the induction of obesity, the difference in the amount (g) of food intake between the two groups––control and diet-induced obesity control––was 17%. An average rat from the control and diet-induced obesity control group ate 23 g and 19 g of food for 24 hours, respectively. If calculated as the calories obtained from food, the difference was significant, i.e. 63% (P<0.05). The difference in the amount of water intake was 14.83%. The results are shown in Table 2.

### The effect of guanfacine on body weight in rats with high-fat diet-induced obesity

After 30 days of treatment with guanfacine at the dose of 0.5 mg/kg b.w./day, the body weight of rats increased by 2.84% in comparison with the body weight measured before the first administration of the drug. After 30 days of treatment, the difference in body weight between the diet-induced obesity control group and the group treated with guanfacine at the dose of 0.5 mg/kg b.w./day was 9.22% (P<0.05), but the differences in body weight changes were significant only on the 18^th^ day of treatment. There was no statistical difference between the controls (receiving standard food) and this tested group after 30 days of treatment. It can be concluded that guanfacine at this dose showed a minor anorectic activity. The changes in body weight are shown in Fig 1.

After 30 days of treatment of the obese rats with guanfacine at the dose of 2 mg/kg b.w./day, the body weight of animals was 15.24% lower than the body weight measured before the first administration of the drug. After 30 days of treatment, the difference between the body weight in the diet-induced obesity control group and the group treated with guanfacine at the dose of 2 mg/kg b.w./day was 22.21% (P<0.001). It can be assumed, however, that guanfacine at this dose possesses a significant anorectic activity. There was no statistically significant difference between the controls (receiving standard food) and this tested group after 30 days of treatment. The changes in body weight are shown in Fig 1.

### The effect of yohimbine on body weight in rats with high-fat diet-induced obesity

After 30 days of treatment of obese rats with yohimbine at the dose of 2 mg/kg b.w./day, the body weight of animals was 8.5% higher than the body weight measured before the first administration of the drug. After 30 days of treatment, the difference between the body weight in the diet-induced obesity control group and the group treated with yohimbine at the dose of 2 mg/kg b.w./day was 3.38% and this was considered insignificant. Even though the rats in this group gained less weight than those in the diet-induced obesity control group, there was no statistical difference between these groups, whereas the difference between the controls (receiving standard food) and the group treated with yohimbine at the dose of 2 mg/kg b.w./day was significant (P<0.001). It can be assumed that yohimbine at this dose did not show a significant anorectic effect. The changes in body weight are shown in Fig 1.

After 30 days of treatment of obese rats with yohimbine at the dose of 5 mg/kg b.w./day, the body weight of animals was 4.09% higher than the body weight measured before the first administration of the drug. After 30 days of treatment, the difference between the body weight in the diet-induced obesity control group and the group treated with yohimbine at the dose of 5 mg/kg b.w./day was 8.17% (P<0.05). The animals treated with yohimbine gained less weight on the 18th and 21st day of the treatment in comparison with the diet-induced obesity control group, but there was subsequently no difference between these groups. Taking into consideration the fact that during 30 days of treatment there was no statistical difference between the controls and the group treated with yohimbine at the dose of 5 mg/kg b.w./day, it can be concluded that yohimbine at this dose showed a minor anorectic effect. The changes in body weight are shown in Fig 1.

### The effect of guanfacine and yohimbine on food and water intake

There was a significant reduction in calorie intake in the group treated with guanfacine at the dose of 2 mg/kg b.w./day (P<0.01) and the group treated with yohimbine at the dose of 5 mg/kg b.w./day (P<0.01) in comparison with the diet-induced obesity control group. Moreover, a significant increase in water consumption in the group treated with guanfacine at the dose of 2 mg/kg b.w./day (P<0.01) and a reduction in the group treated with yohimbine at the dose of 5 mg/kg b.w./day (P<0.05) were observed. The results are shown in Table 3.

**Table 3:** The effect of studied compounds on body weight, food and water intake.

	Diet-induced obesity control	Yohimbine 2 mg/kg b.w.	Yohimbine 5 mg/kg b.w.	Guanfacine 0.5 mg/kg b.w.	Guanfacine 2 mg/kg b.w.
**Total food intake, kcal**	2532.2 ± 28.76	2218.2 ± 204.33	1945.3 ± 106.32	2081.2 ± 189.53	1676.9 ± 155.21
**Total food intake, g**	460.4 ± 5.23	403.3 ± 37.15	353.7 ± 19.33^##^	378.4 ± 34.46	304.9 ± 28.22^##^
**Total water intake, ml**	831.9 ± 47.50	1041.3 ± 169.70	695.0 ± 40.21^#^	904.3 ± 77.78	1288.8 ± 107.10^##^

Food and water intake during 30 days treated using the test compounds in Wistar rats on diet-induced obesity; Treatment: Diet-induced obesity control group—water; Yohimbine at 2 mg/kg b.w., i.p.; Yohimbine at 5 mg/kg b.w., i.p.; Guanfacine at 0.5 mg/kg b.w., i.p.; Guanfacine at 2 mg/kg b.w., i.p.;

^#^Significant vs. diet-induced obesity control group, P<0.05;

^##^Significant vs. diet-induced obesity control group, P<0.01.

### The effect of guanfacine and yohimbine on the amount of intraperitoneal adipose tissue

Guanfacine injected intraperitoneally at the doses of 0.5 or 2 mg/kg b.w. for 30 days significantly reduced the amount of intraperitoneal adipose tissue in comparison with the diet-induced obese control group (P<0.01). Similarly, yohimbine administered for 30 days at the dose of 5 mg/kg b.w. showed the same effect (P<0.01). There were no significant changes between the amount of intraperitoneal adipose tissue in these groups and in the control group (receiving standard feed). The effect of compounds on the amount of intraperitoneal adipose tissue is shown in Table 4.

**Table 4:** The effect of studied compounds on the amount of intraperitoneal adipose tissue.

	Control	Diet-induced obesity control	Yohimbine 2 mg/kg b.w.	Yohimbine 5 mg/kg b.w.	Guanfacine 0.5 mg/kg b.w.	Guanfacine 2 mg/kg b.w.
**Weight of intraperitoneal fat tissue [g]**	15.5 ± 2.56	28.5 ± 2.28**	23.0 ± 1.49*	14.9 ± 1.30^##^	17.5 ± 1.14^##^	14.2 ± 2.59^##^
**Percentage of body weight**	3.90	6.17	5.14	3.49	4.13	3.95

Weight of intraperitoneal fat tissue after 30 days treated using the test compounds in Wistar rats on diet-induced obesity. Treatment: Diet-induced obesity control group—water; Yohimbine at 2 mg/kg b.w., i.p.; Yohimbine at 5 mg/kg b.w., i.p.; Guanfacine at 0.5 mg/kg b.w., i.p.; Guanfacine at 2 mg/kg b.w., i.p.;

*Significant vs. control group, P<0.05;

**Significant vs. control group, P<0.01;

^##^Significant vs. diet-induced obesity control group, P<0.01.

### The effect of chronic 30-day treatment with guanfacine and yohimbine on lipid and carbohydrate profiles and glycerol levels in rats fed a high-fat diet

**Fig 2:**
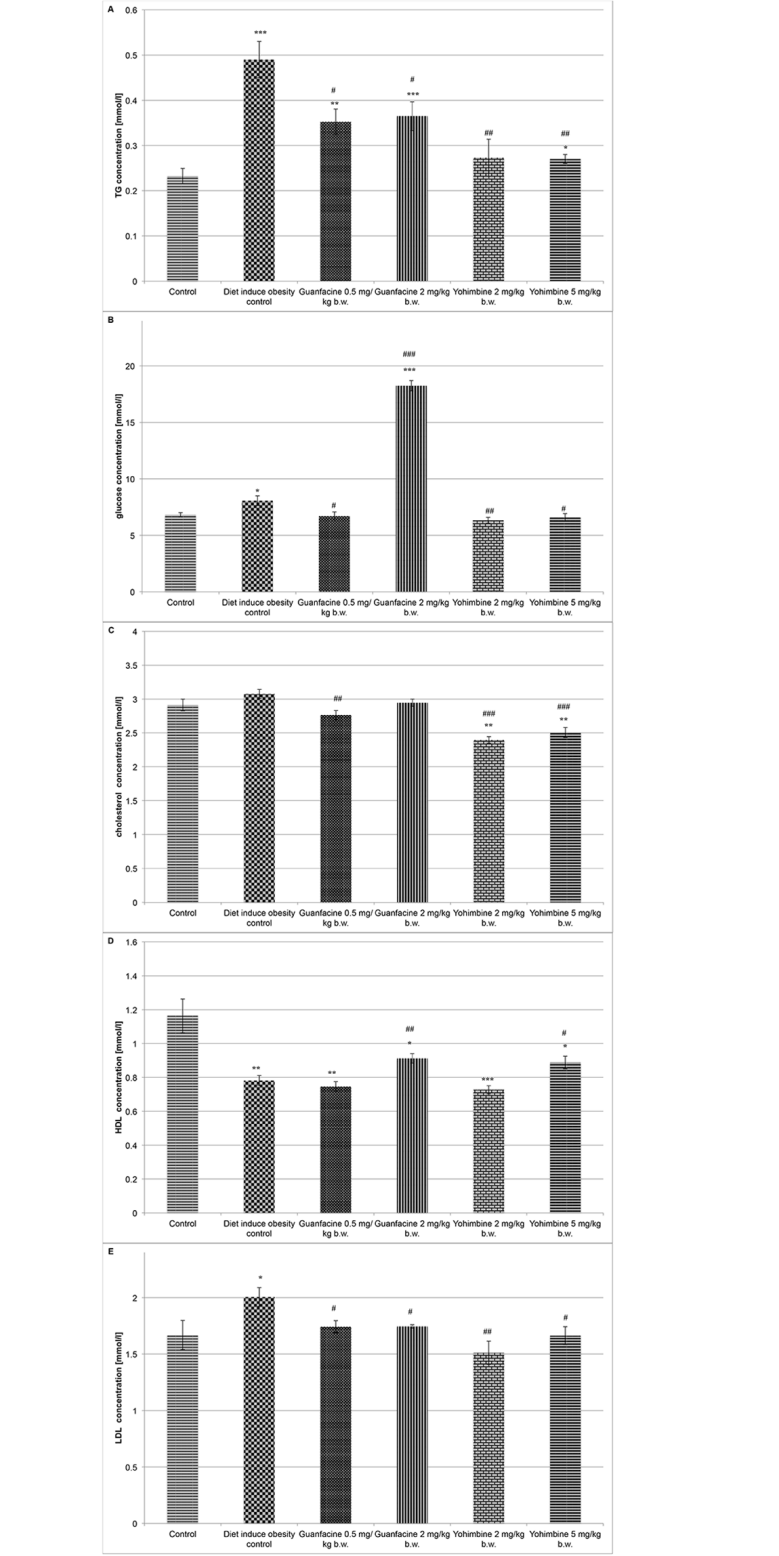
The effect of treatment with guanfacine and yohimbine on lipid and carbohydrate profile. **A**. TG; **B**. Glucose; **C**. Total cholesterol; **D**. HDL-cholesterol; **E**. LDL-cholesterol; Control group—water; Diet-induced obesity control group—water; Yohimbine at 2 mg/kg b.w., i.p.; Yohimbine at 5 mg/kg b.w., i.p.; Guanfacine at 0.5 mg/kg b.w., i.p.; Guanfacine at 2 mg/kg b.w., i.p.; *Significant vs. control group, #—significant vs. diet-induced obese control group: *P<0.05, **P<0.01, ***P<0.001 (t-Student).

The 30-day treatment with yohimbine and guanfacine significantly improved lipid profiles and decreased the level of triglycerides in plasma in rats fed with high-fat feed. Moreover, both yohimbine at the doses of 2 mg/kg b.w. and 5 mg/kg b.w., and guanfacine at the dose of 0.5 mg/kg b.w. significantly decreased plasma glucose levels in these animals. In contrast, guanfacine at 2 mg/kg b.w./day significantly increased plasma glucose levels. These results are shown in Fig 2. Both guanfacine at the dose of 2 mg/kg b.w. and yohimbine at the dose of 5 mg/kg b.w. significantly increased glycerol levels in plasma in comparison with the levels of glycerol observed in the plasma of obese control rats. The results (mean ± SEM) were: diet-induced obese control group 21.1 ± 1.28; guanfacine 28.4 ± 1.81 (t-Student test, **P<0.01); yohimbine 33.3 ± 2.51 (t-Student test, **P<0.01).

### The effect of intraperitoneal administration of studied compounds on blood pressure and heart rate in normotensive rats

**Fig 3:**
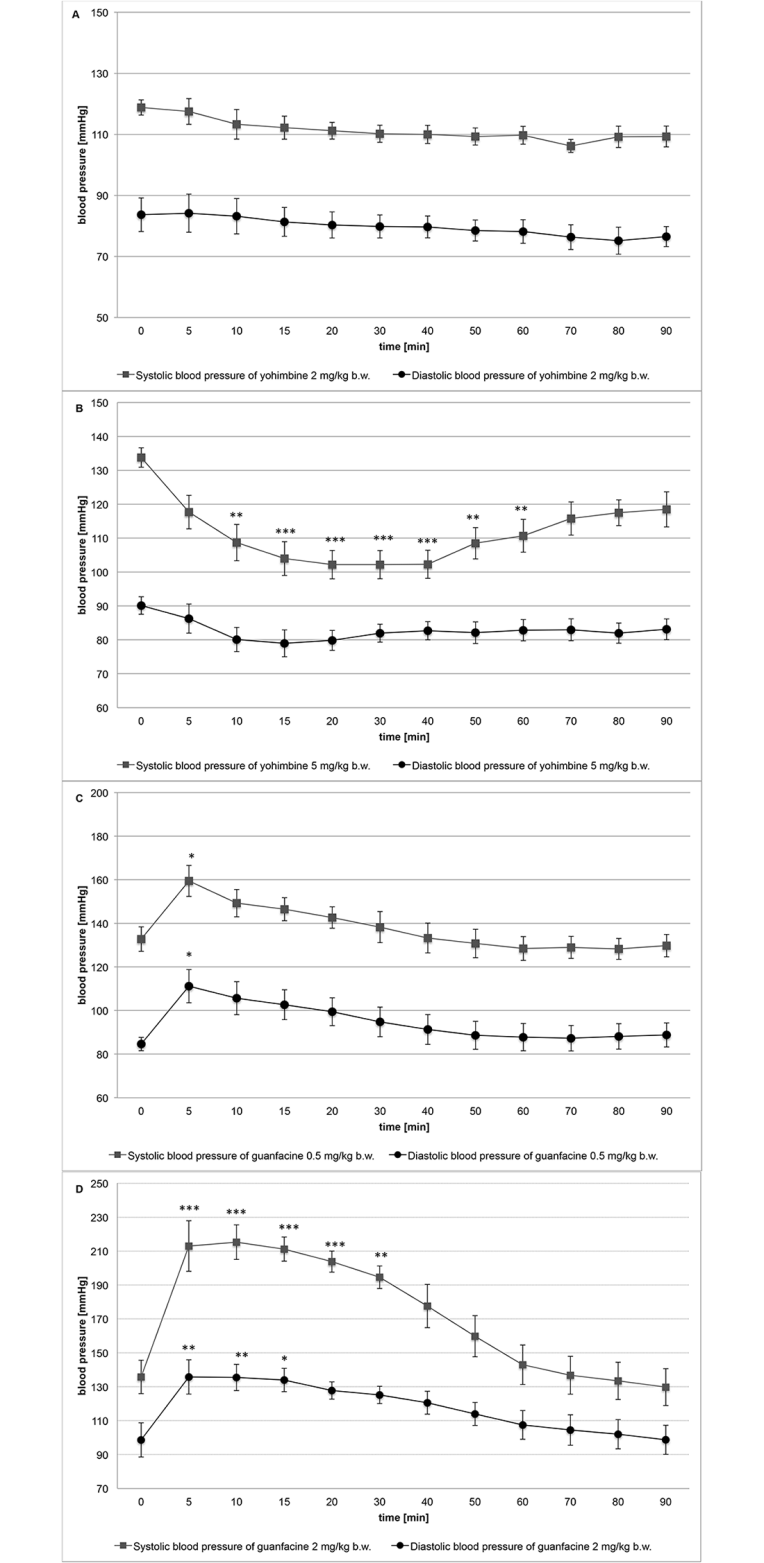
The effect on blood pressure after acute treatment with the studied compounds. **A**: Yohimbine at 2 mg/kg b.w., i.p.; **B**: Yohimbine at 5 mg/kg b.w., i.p.; **C**: Guanfacine at 0.5 mg/kg b.w., i.p.; **D**: Guanfacine at 2 mg/kg b.w., i.p. Mean ± SEM; Significant to time 0: *P<0.05, **P<0.01, ***P<0.001, (ONE-WAY ANOVA)

Guanfacine administered intraperitoneally at the dose of 2 mg/kg b.w. significantly increased blood pressure and caused significant bradycardia. Acute treatment with a dose of 0.5 mg/kg b.w., i.p. induced a significant increase in blood pressure, but only in the 5th minute after the administration. Yohimbine administered intraperitoneally at the dose of 5 mg/kg b.w. significantly lowered blood pressure and decreased heart rate, whereas at the dose of 2 mg/kg b.w., i.p. it had no significant effect on blood pressure. The results are shown in Fig 3 and Table 5.

**Table 5:** Changes in heart rate during 90 minutes after intraperitoneal administration of the studied compounds.

**A**	time	0	5	10	15	20	30	40	50	60	70	80	90
	mean	281.5	299.8	280.2	261.8	254.2	267.5	260.5	254.7	262.0	286.5	269.2	267.2
	SEM	26.49	29.22	37.20	35.95	41.40	31.92	37.21	36.91	27.53	30.62	24.6	24.27
**B**	time	0	5	10	15	20	30	40	50	60	70	80	90
	mean	324.0	312.7	284.0	268.8	259.3	255.0	251.5	256.5	251.0	256.5	261.5	246.0
	SEM	12.48	15.07	8.97	7.21	6.58	8.20	13.71	18.62	22.35	18.50	21.06	18.72
						*	*	*	*	*	*	*	*
**C**	time	0	5	10	15	20	30	40	50	60	70	80	90
	mean	275.8	270.3	259.3	247.3	238.0	226.0	215.8	209.0	201.8	194.8	194.8	191.5
	SEM	23.12	7.48	6.70	6.85	6.31	6.06	6.84	6.40	7.17	6.80	6.80	6.91
							**	***	***	***	***	***	***
**D**	time	0	5	10	15	20	30	40	50	60	70	80	90
	mean	354.0	286.0	227.8	267.0	264.2	249.0	249.2	236.5	221.3	233.3	230.8	231.8
	SEM	8.63	17.40	11.95	11.18	13.88	19.44	18.39	22.67	28.29	23.97	24.29	26.38
			*	*	**	**	**	***	***	***	***	***	***

**A** Yohimbine 2 mg/kg b.w.; **B** Yohimbine 5 mg/kg b.w.; **C** Guanfacine 0.5 mg/kg b.w.; **D** Guanfacine 2 mg/kg b.w.; Signifcant vs. 0 time

*P<0.05;

**P<0.01;

***P<0.001, ONE-WAY ANOVA

### The effect of studied drugs on locomotor activity during chronic treatment in diet-induced obese rats housed in pairs in home cages

**Fig 4:**
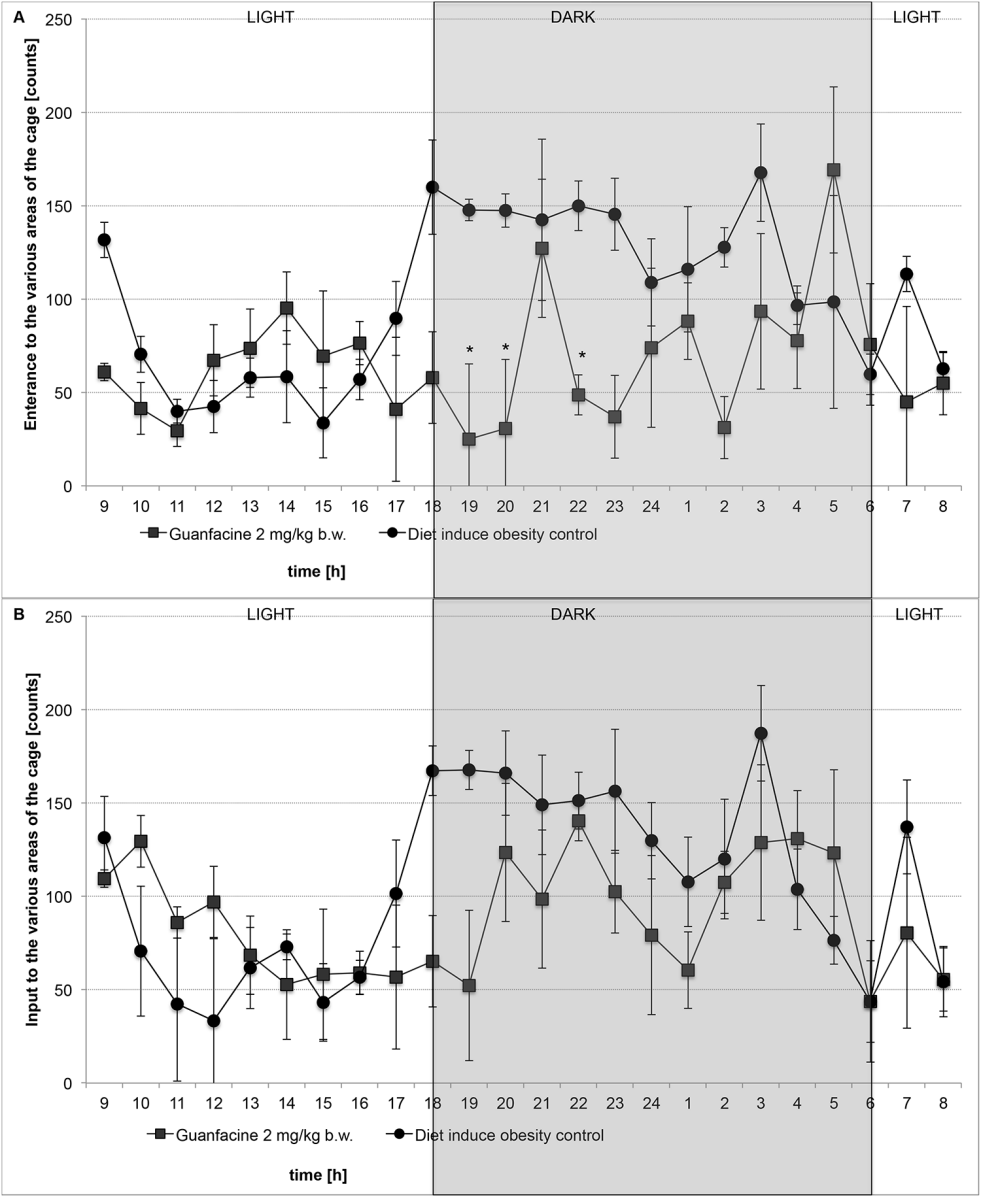
The effect on locomotor activity after treatment with guanfacine in diet-induced obese rats. After first administration (A) or after 29th administration (B): Diet-induced obesity control group—water or Guanfacine at 2 mg/kg b.w., i.p.; Mean ± SEM; Significant to diet-induced obese control group: * P<0.05 (TWO-WAY ANOVA)

Guanfacine at the dose of 2 mg/kg b.w. significantly decreased locomotor activity after single administration in diet-induced obese rats in comparison with the diet-induced obesity control group. However, after 29 days of administration of the compound, there was no statistical significance. The results are shown in Fig 4.

### The effect of guanfacine or yohimbine on core temperature in rats

**Fig 5:**
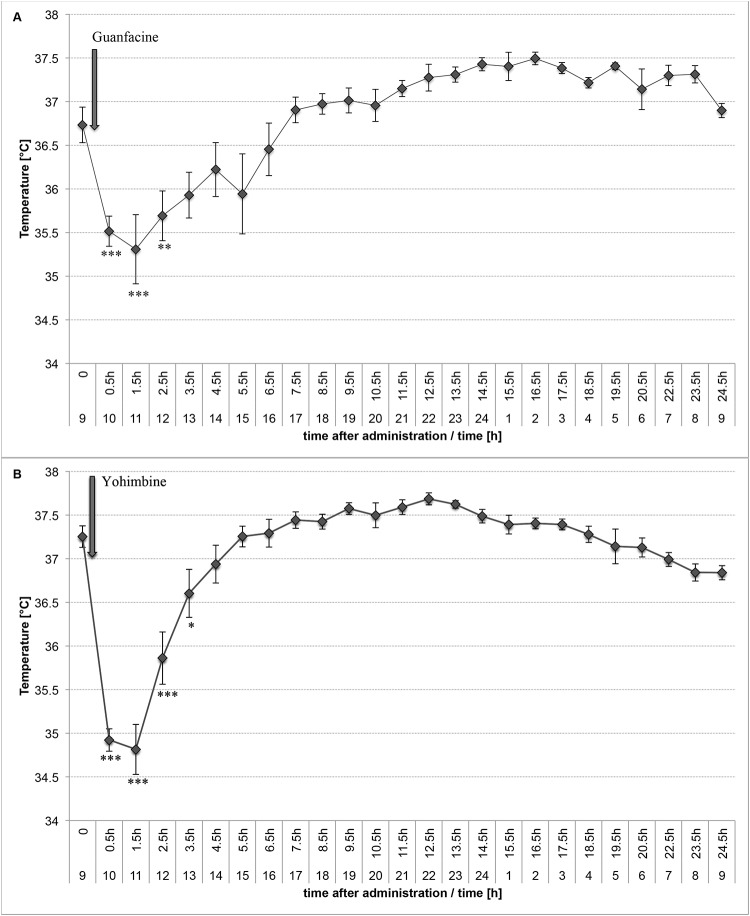
The effect on core temperature after treatment with guanfacine (A) or yohimbine (B). The changes in core temperature after a single, intraperitoneal administration of (A) Guanfacine at 2 mg/kg b.w.; (B) Yohimbine at 5 mg/kg b.w.; Mean ± SEM; Significant to time 0 = 0.5 hour before administration compound 1 (9.15am): * P<0.05, **P<0.01, ***P<0.001, (ONE-WAY ANOVA)

Both guanfacine and yohimbine significantly decreased core temperature in rats. The effect was significant for three hours, and after this time we observed an increase in the temperature. This may be a result of the rapid activation of the mechanisms responsible for maintaining the temperature homeostasis at the appropriate level. The results are shown in Fig 5. Furthermore, in rats given guanfacine we observed significant sweating, which may suggest that the thermogenesis was intensified.

### The effect of chronic 30-day treatment with guanfacine and yohimbine on cortisol levels in plasma of rats fed a high-fat diet

In obese rats, yohimbine administered for 30 days showed no effect on the level of cortisol in plasma. In this group, the cortisol plasma level was comparable to that in the control group (mean ± SEM: t-Student test, 105.4 ± 2.27 for the group treated with yohimbine at 5 mg/kg b.w./day and 108.0 ± 1.51 for the diet-induced obesity control group) and the glucose level was significantly lower in comparison with the control group. Given the above data, we can exclude stress as a factor responsible for weight loss after administration of yohimbine. In contrast, guanfacine administered at a dose of 2 mg/kg b.w. for 30 days significantly increased cortisol levels in plasma (mean ± SEM: Student's t-test, 130.3 ± 1.57 for the group treated with guanfacine at the dose of 2 mg/kg b.w./day, *** P<0.001) compared with the level in the diet-induced obesity control group. Moreover, it also significantly raised the level of glucose. Unfortunately, the above results show that this compound at this dose intensified stress in animals.

### Lipolysis *in vitro*

In contrast with guanfacine, yohimbine did not induce a cytotoxic effect at a concentration of 50 μM, as confirmed by a viability assay (PrestoBlue). This assay is an indicator of mitochondrial metabolic activity, particularly the mitochondrial electron chain. A decrease in PrestoBlue fluorescence is an indicator of mitochondrial damage or inactivation of cytosolic and microsomal enzymes. The tested compounds did not exhibit cytotoxic activity in the Toxilight test, which measures the final stages of cellular death. The test compounds did not affect the process of lipolysis, but at a concentration of 1 μM they increased the glucose utilization by adipocytes. The results are summarized in Table 6.

**Table 6:** The effect of the tested compounds on cell viability, cell membrane damage, glucose utilization and lipolysis.

Compound	Concentration of the compound	Cell viability	Cell membrane damage	Glucose utilization	Lipolysis
**Yohimbine**	50 μM	94.0 ± 2.99	95.3 ± 6.60	95.6 ± 5.60	100.0 ± 2.99
	10 μM	103.7 ± 1.46	102.7 ± 2.04	101.3 ± 1.63	100.0 ± 4.48
	1 μM	97.0 ± 2.25	109.7 ± 1.47	118.1 ± 0.36 ***	99.3 ± 4.97
**Guanfacine**	50 μM	82.7 ± 0.72 ***	95.0 ± 8.47	102.0 ± 3.51	100.0 ± 3.53
	10 μM	103.0 ± 1.40	92.0 ± 0.65	103.9 ± 0.58	100.0 ± 1.82
	1 μM	99.7 ± 3.19	104.7 ± 6.86	112.1 ± 4.76 **	103.0 ± 4.08

% of control, mean ± SEM;

**P<0.01;

***P<0.001, ONE-WAY ANOVA

## Discussion

Guanfacine is a long-acting [13], partial α_2_-adrenoceptor agonist [11]. It activates α_2A_-adrenoceptors located mainly presynaptically [14]. Guanfacine also has the ability to activate α_1_-adrenoceptors [15]. It has been approved by FDA to treat hypertension, and lately to treat conduct disorder in children and adolescents [10, 16].

Our study did not confirm the safety of guanfacine in obesity when it was administrated at 2 mg/kg b.w.. Unfortunately, the weight reduction effect caused by guanfacine should be considered as a symptom of side effects (enhanced stress, decreased spontaneous motility, abnormal blood pressure and heart rate). However, since there was a significant decrease in body weight in rats with induced obesity including at a lower dose that does not produce such undesirable effects, the mechanisms of this phenomenon should be explored.

Our study confirms that guanfacine is a partial α_2A_- and α_2B_-adrenoreceptor agonist. It can, therefore, reduce the release of norepinephrine into the synaptic cleft, thus inhibiting the physiological response to the postsynaptic receptors. However, because it is also a partial agonist, the stimulation of α_2_-adrenoreceptors is maintained at a certain level (lower than at the physiological stimulation by noradrenalin). Since guanfacine is a long-acting agonist [13], it occupies adrenoreceptors preventing their rapid stimulation by successive portions of noradrenaline, which certainly reduces the stimulating action of noradrenaline at α_2_- and α_1_-adrenoceptors, but can also lead to increased noradrenaline binding to β-adrenoreceptors.

Our studies on intrinsic activity confirm that yohimbine is a non-selective antagonist of α_2_-adrenoceptors. Some studies suggest that it acts preferentially through α_2A_-adrenoceptors, blocking their activation both pre- and postsynaptically [14]. At higher doses, yohimbine also acts as an α_1_-adrenoceptor antagonist. By blocking presynaptic α_2A_-adrenoceptors, yohimbine increases the release of noradrenaline into the synaptic cleft, but it also blocks postsynaptic α_2A_-adrenoceptors and, to a lesser extent, α_2B_- and α_1_-adrenoceptors. Therefore, after its application the released noradrenaline mainly stimulates β-adrenoceptors, but also to a lesser extent α_2B_- and α_1_-adrenoceptors (depending on dose). These actions are undoubtedly related to its weight reducing action.

Our research has shown that yohimbine as well as guanfacine showed weight reducing and anorectic action in a high-fat diet-induced obesity model in rats. Guanfacine shows a much stronger effect. The effect of yohimbine after administration at 2 mg/kg b.m. was not significant, while guanfacine reduced weight at as low a dose as 0.5 mg/kg b.m.. The analysis of the data concerning the effect on adrenergic receptors shows that this effect is caused by partial activation of postsynaptic α_2B_- and α_1_-adrenoceptors. Another important fact is that both drugs decrease physiological activation of α_2A_-adrenoceptors and increase physiological activation of β-adrenoceptors by noradrenaline. It can be concluded that to achieve a weight reducing effect, it is essential for drugs acting on adrenergic receptors to reduce, even partially, the response of α_2A_-adrenoceptors, and to maintain partial activity of α_2B_-adrenoceptors.

This is in agreement with the results obtained by Janhunsen et al., who showed that by activating α_1_-, α_2B_- and β_2_-adrenoreceptors noradrenaline reduces calorie intake, and by the activation of α_2A_-adrenoceptors increases it [8].

Temperature regulation in brown adipocyte tissue is mediated by the adrenergic system. Adipose tissue is highly innervated by sympathetic nerves, which activate stimulation when exposed to cold and food. Since the activation of α_2_- and β_3_-adrenoceptors causes opposite effects, the α_2_/β_3_adrenoreceptor ratio is very important not only in thermogenesis but also in the control of lipolysis in white adipose tissue [17]. The thermogenic action of α_2_-adrenoceptor agonists leads to a reduction of thermogenesis [18, 19]. Guanfacine acts in an opposite manner, i.e. anti-thermogenically [13], which is connected with increasing thermogenesis. In our study, guanfacine significantly reduced body temperature. Furthermore, we observed intense sweating in animals which were treated with this drug. This indicates that guanfacine may increase thermogenesis, which consequently causes weight reduction. It is possible, though, that treatment with a partial agonist such as guanfacine reduces the response of natural agonists, and causes effects opposite to those of agonists, i.e. antithermogenic and lipolytic effects.

Guanfacine is not a compound which acts typically like other α_2_-adrenoreceptor agonists, such as clonidine or xylazine [13, 20]. It has been proven in mice that guanfacine administered intragastrically at the doses of 0.5 and 2 mg/kg b.w. caused a dose-dependent decrease in appetite and reduced weight gain compared with control animals [13]. In contrast, clonidine induced an increase in food intake in rats [21]. Furthermore, α_2_-adrenoreceptor agonists cause thermogenic action [19], and guanfacine antithermogenic [13]. Moreover, α_2_-adrenoreceptor agonists inhibit lipolysis [22], and reduce insulin levels by inhibiting its release [23, 24], which leads to impaired glucose tolerance and its increased level in blood [24]. Guanfacine causes adipose tissue loss [13], which was also observed in our studies.

It is common knowledge that a decrease in food intake leads to increased lipolysis and hence to the hydrolysis of triglycerides into free fatty acids and glycerol in adipocyte lipid droplets [25]. In our studies, guanfacine significantly reduced food intake and caused a decrease in plasma triglyceride and glycerol levels. Therefore, the inhibitory effect of guanfacine on appetite may also be associated with increased lipolysis. Weight reduction after guanfacine treatment begins very quickly. In our studies, the body weight of rats was lower from the first day of guanfacine administration, and during the next 30 days it remained on a negative balance. In obese rats, the number of α_2_-adrenoceptors and the antilipolytic activity in adipocytes is increased [23]. Because α_2_- and β_3_-adrenoceptors coexist on the same fat cell, the ratio of functional α_2_- and β_3_-adrenoceptors present in adipose tissue may determine whether fat storage or release is activated by catecholamines and, therefore, this may play a significant role in regulating energy balance [26]. Partially blocking the response of noradrenaline at the excessive amount of α_2_-adrenoceptors, after treatment with guanfacine, may lead to the activation of lipolysis, because the response of α_2_-adrenoceptors is decreased and noradrenaline mainly stimulates β-adrenoceptors. *In vitro* studies have not confirmed the effect of guanfacine on lipolysis. However, we have reported a significant increase in plasma glycerol levels in animals treated with this drug, which suggests that guanfacine may show a lipolytic activity *in vivo*.

The reason for increased levels of glucose after guanfacine treatment may be the rapid and large increase in the release of free fatty acids from adipocytes which reach the liver in too large quantities, and interfere with its function, which causes glucose intolerance [27]. Some studies suggest that there is a significant improvement in glucose tolerance and only slight one in insulin secretion in patients with hypertension and diabetes, when treated with guanfacine for one year [28]. There are also divergent data concerning the effect of guanfacine on plasma cholesterol levels [29, 30].

Cortisol is a stress hormone produced by the adrenal glands and is commonly measured to assess stress [31]. To exclude the possibility that weight loss could be the effect of stress associated with chronic administration of the drugs, we determined cortisol levels in plasma. In chronic stress an increase in e.g. corticotropin, cortisol, vasopressin and thyroxin is observed. The increase in cortisol is connected with a subsequent increase in blood glucose levels and an increase in appetite. Unfortunately, the plasma cortisol level in animals treated with guanfacine at the dose of 2 mg/kg b.w. was significantly higher than in the control group. Additionally, an increase in glucose levels was noticed. These factors clearly demonstrate that guanfacine at this dose causes stress and its weight reducing properties may be associated with this.

Undoubtedly, the important action against obesity after chronic administration of yohimbine is the compensation of impaired lipid profiles and lowering glucose levels, which was confirmed in our study. These favorable properties are probably related to the blockade of α_1_-adrenoceptors by this drug. It is well known that α_1_-adrenoceptor antagonists act positively on lipid and carbohydrate profiles [32].

It has been noticed that, in individuals with an increased risk of developing obesity and diabetes, there are too many α_2A_-adrenoceptors in beta cells in the pancreas, which impairs proper insulin release and leads to hyperglycemia [33]. Undoubtedly, blocking these receptors by yohimbine may also lead to a reduction in plasma glucose levels.

The fact is that guanfacine does not block α_1_-adrenoreceptors, and moreover, at higher doses it stimulates them. The similar effects on lipid profile after yohimbine and guanfacine cannot be explained taking into consideration only their effect on this type of adrenergic receptor. There are also divergent data concerning the effect of guanfacine on plasma cholesterol levels [34, 35]. In the world’s literature there are equivocal studies concerning the effect of α_1_-adrenoreceptor agonists on lipid and carbohydrate profiles. There are some reports that xylazine induces an increase in plasma glucose level by α_1_-adrenoreceptor activation [18]. The exact mechanism by which guanfacine causes hyperglycemia has not yet been determined. Our present study suggests that this may be connected with stress caused by this drug.

Yohimbine at the dose acting anorexically (5 mg/kg b.w.) showed a hypotensive effect. This correlates with the findings that the drug blocks α_2_-adrenoreceptors, and in large doses also α_1_-adrenoreceptors. Non-selective α_2_-adrenoreceptor antagonists possess hypotensive properties, because they cause vasodilation by blocking α_2B_-adrenoreceptors and prevent vasospasm by blocking α_2C_-adrenoreceptors [22].

The effect of guanfacine on blood pressure confirms that it can activate α_2B_-adrenoceptors, and also α_1_-adrenoceptors. Thus, the regulation of blood pressure at the periphery is regulated via these receptors [36]. It has been proven that non-selective activation of α_2_-adrenoceptors usually leads to a biphasic response in blood pressure: after the short-term pressure increase phase, the pressure falls below baseline. The activation of α_2B_-adrenoceptors is responsible for the increase in blood pressure, while the activation α_2A_-adrenoceptors induces a long-lasting phase of drop in blood pressure [22].

Statistically significant bradycardia shown in this study after administration of guanfacine can confirm the reduction in the response of β_1_-adrenoceptors to noradrenaline, and it can be the result of stimulation of the baroreceptor reflex in response to a strong hypertensive effect in the initial period after administration. In the literature, there are reports of significant, long-lasting, hypertensive response and long-term bradycardia in patients who have taken guanfacine in large doses [36].

Guanfacine decreased spontaneous activity in the first week of treatment. This effect may be connected with the reduction of weight. This is an alarming effect of this drug. In the fourth week, no significant difference in the activity between the studied group and the obese rats in the control group was observed, which indicates that tolerance to sedation occurs after chronic administration of guanfacine. Reduced tolerance can also be caused by abnormalities in blood pressure and heart rate, and consequently the malaise of animals in the first week of treatment.

There is a risk associated with the chronic use of α_2A_-adrenoreceptor antagonists. It is well known that a long-term blockade of the receptors leads to the up-regulation phenomenon—an increase in their number and sensitivity to physiological agonists [37]. The effectiveness of the antagonists may therefore be short-term. The reduction of receptor response without the risk of up-regulation is theoretically possible by using compounds with partial intrinsic activity, which stimulate the receptor but significantly reduce the response of natural agonists.

## Conclusion

This study for the first time clearly indicates the effective anorectic action of guanfacine in obese rats, and shows that such an effect can be achieved by partial α_2A/B_-adrenoreceptor agonists. This is the first report that partial α_2_-adrenoreceptor agonists may possess body weight reducing activity in the model of obesity.

Yohimbine is less effective in reducing body weight than guanfacine, but it improves lipid and carbohydrate profiles, and it does not cause such serious side effects.

We believe, however, that α_2_-adrenoceptor ligands should still be considered potential drugs reducing body weight, but we should rather look for partial agonists, which will not show side effects similar to guanfacine and yohimbine.

We suggest, that partial agonists of α_2A/B_-adrenoreceptors with a very weak intrinsic activity seem to offer a potential for a good and effective treatment of obesity, with a lower risk of side effects associated with the cardiovascular system. Of importance is also the lack of affinity for α_1_-adrenoceptors. These properties will certainly reduce the occurrence of side effects. However, it should be emphasized that both the blockade and activation α_1_-adrenoceptors may improve therapeutic effects, because the activation leads to a decrease in calorie intake, and the blockade to the improvement of lipid and carbohydrate profiles.
